# Advanced glycation end product accumulation and AGEs–RAGE axis biomarkers in plasma and synovial fluid are associated with osteoarthritis severity

**DOI:** 10.17305/bb.2026.14274

**Published:** 2026-06-22

**Authors:** Anja Šoštarič, Iza Oblak, Dušan Šuput, Irina Milisav, Blaž Mavčič

**Affiliations:** 1 Department of Orthopedic Surgery, University Medical Centre Ljubljana, Ljubljana, Slovenia; 2 Chair of Orthopedics, Faculty of Medicine, University of Ljubljana, Ljubljana, Slovenia; 3 Institute of Pathophysiology, Faculty of Medicine, University of Ljubljana, Ljubljana, Slovenia; 4 Laboratory of Oxidative Stress Research, Faculty of Health Sciences, University of Ljubljana, Ljubljana, Slovenia

**Keywords:** Osteoarthritis, degenerative joint disease, skin autofluorescence, synovial fluid, AGE receptor, RAGE

## Abstract

Osteoarthritis (OA) is a common whole-joint degenerative disease characterized by cartilage degradation, subchondral bone remodeling, synovial inflammation, and incompletely understood molecular mechanisms. Advanced glycation end products (AGEs) may contribute to OA pathophysiology by altering extracellular matrix properties and activating inflammatory signaling through the receptor for advanced glycation end products (RAGE). This study investigated the association between AGE accumulation and OA severity and evaluated AGE-related biomarkers in plasma and synovial fluid. In this cross-sectional observational study with prospective recruitment and retrospective clinical and radiographic assessment, skin AGE accumulation was measured by skin autofluorescence (SAF) in 404 outpatients aged 55–75 years. Lower-limb OA burden was assessed using clinical and available radiographic data for hip and knee OA, while hand osteoarthritis (HOA) was evaluated clinically. In a surgical subgroup of 42 participants, plasma AGEs, assessed as Nɛ-(carboxymethyl)lysine (CML), and soluble RAGE (sRAGE) concentrations were measured using enzyme-linked immunosorbent assay (ELISA). Paired plasma and synovial fluid analyses were performed in 21 OA patients with available synovial fluid samples. Higher SAF was independently associated with greater lower-limb OA severity after adjustment for age, sex, body mass index (BMI), and smoking status [odds ratio (OR) 4.06, 95% confidence interval (CI) 2.54–6.46; *P <* 0.001]. SAF was also strongly associated with HOA (OR 15.0, 95% CI 7.29–30.91; *P <* 0.001). In the biochemical subgroup, SAF inversely correlated with synovial sRAGE (*ρ ═* −0.571; *P ═* 0.007), and plasma AGEs inversely correlated with plasma sRAGE (*ρ ═* −0.481; *P ═* 0.027). Both AGEs and sRAGE concentrations were lower in synovial fluid than in plasma. These findings support an association between systemic AGE accumulation and OA burden and suggest that altered AGEs–RAGE axis activity may contribute to OA pathophysiology. Further longitudinal studies are needed to clarify whether SAF may serve as a non-invasive biomarker of OA burden.

## Introduction

Osteoarthritis (OA) is the most prevalent degenerative joint disease, characterized by progressive articular cartilage degeneration, subchondral bone remodeling, and synovial inflammation, which collectively lead to chronic pain and impaired joint function [1, 2]. Compared to healthy joints, OA is associated with cartilage degradation, subchondral bone thickening, osteophyte formation, and structural and inflammatory changes in the synovial tissue and meniscus [2].

As OA progresses, structural and functional alterations occur across multiple joint tissues. Recent evidence also highlights the potential role of the infrapatellar fat pad in OA progression, including fibrotic and biomechanical changes that may contribute to local joint environment and joint mechanics alterations [3]. These changes negatively impact quality of life and often necessitate surgical intervention. Although joint replacement remains the standard treatment for advanced disease, it does not address the underlying pathogenic mechanisms of OA. While OA is readily diagnosed through clinical and radiographic assessments, its underlying disease mechanisms remain incompletely understood. Therefore, current research increasingly focuses on identifying the pathogenic mechanisms involved in OA development and progression [1–4].

Several risk factors are known, including age, obesity, hormonal influences, genetic predisposition, mechanical stress, occupational exposure, previous injury, and systemic disease; however, the cellular and molecular pathogenesis is only partially understood [3, 4].

This complexity is compounded by associations with systemic diseases. The biochemical properties of cartilage depend on the integrity of its extracellular matrix (ECM), which primarily consists of collagen and proteoglycans. In OA, these components gradually degrade, impairing joint function. OA cartilage also exhibits altered biomechanical properties, including impaired responses to mechanical loading and reduced ability to maintain tissue homeostasis. Additionally, destabilization of the collagen network and altered structure of matrix molecules, coupled with the cellular response, significantly contribute to degeneration [5]. Meniscal dysfunction is predominantly degenerative, although inflammation, such as synovial fluid secretion, can exacerbate it [6].

Advanced glycation end products (AGEs) contribute to the heterogeneity of matrix molecules [7]. AGEs are a heterogeneous and chemically diverse group of compounds formed either endogenously or exogenously through various pathways in the human body. They are formed non-enzymatically via a condensation reaction between carbonyl groups of reducing sugars and free amine groups of proteins, lipids, or nucleic acids (Maillard reaction), followed by further molecular rearrangements that yield stable and irreversible products. These compounds tend to accumulate in tissues and body fluids, particularly under conditions of hyperglycemia and oxidative stress, and can be either fluorescent or non-fluorescent. AGEs impact tissues by directly altering structural proteins (e.g., reducing elasticity) and by binding to the receptor for advanced glycation end-products (RAGE), triggering oxidative stress, inflammation, and cellular dysfunction [8, 9]. RAGE is a multiligand signaling receptor that exists in several isoforms: the membrane-bound form with signaling function and soluble forms thought to reduce signaling [10–13]. Soluble receptors, or sRAGE, are formed either by alternative splicing or proteolytic cleavage of the membrane-bound receptors [11–13].

AGEs are associated with pathological changes in cartilage and the ECM, including decreased elasticity and increased stiffness, which may contribute to OA development and progression [14]. They have been detected in the serum, synovial fluid, and cartilage of OA patients, although local levels may vary or even decrease depending on the disease stage [15]. Increased AGE accumulation in cartilage tissue has been associated with reduced proteoglycan synthesis in aging individuals, supporting their role in OA pathophysiology [16]. AGEs can affect joint tissue integrity by forming collagen cross-links, reducing elasticity, and increasing stiffness. These modifications are similar to those observed in the cartilage of OA patients and may accelerate tissue degeneration, although a direct causal relationship has not been confirmed [15]. While mechanical factors have long been recognized, there is increasing evidence for the role of biochemical processes such as glycation, which affects chondrocyte function and matrix remodeling. By altering chondrocyte behavior and ECM structure, AGEs may contribute to cartilage degradation and promote OA development and progression [7, 14, 17, 18].

Therefore, this study aimed to investigate the relationship between AGE accumulation and OA severity and to further evaluate AGE-related biomarkers in plasma and synovial fluid.

## Materials and methods

This cross-sectional observational study, featuring prospective participant recruitment and retrospective review of clinical and radiographic data, was conducted at the Department of Orthopedic Surgery, University Medical Centre Ljubljana. The study included consecutive adult patients aged 55–75 years presenting to the hospital and outpatient clinic for outpatient orthopedic evaluation or planned orthopedic surgery, including total knee arthroplasty. All participants provided written informed consent. To minimize variability in skin autofluorescence (SAF) measurements related to skin pigmentation, only individuals of Central European origin were included.

Exclusion criteria, assessed through medical history and review of available medical documentation, included rheumatoid arthritis, chronic kidney disease, diabetes mellitus requiring pharmacological treatment, immunosuppressive therapy, post-traumatic OA, malignancy, visible skin alterations at the measurement site, and other systemic inflammatory or connective tissue diseases.

Demographic and clinical data collected for all participants included age, gender, body mass index (BMI), smoking status (current/former/never), presence of known comorbidities, and medication use.

OA severity for each hip and knee joint was assessed using available clinical and radiographic data. When radiographic imaging was available, OA severity was graded according to the Kellgren–Lawrence (KL) classification system. Joints without radiographic evidence of OA were assigned a score of 0, while joints with mild-to-moderate OA were assigned a score of 1. Severe OA was assigned a score of 2 and was defined either by the presence of total joint replacement or placement on the waiting list for joint replacement surgery due to advanced symptomatic OA.

Radiographic imaging was not routinely performed for study purposes in asymptomatic participants to avoid unnecessary radiation exposure. Consequently, radiographic data were not available for all participants, and a standardized radiographic assessment using the KL classification system could not be consistently applied across the entire study cohort. In participants without available radiographic imaging, the absence of clinically relevant OA was additionally supported by the absence of a documented clinical diagnosis of OA, no history of specialist treatment for OA, and a low symptom burden assessed using the Western Ontario and McMaster Universities Osteoarthritis Index (WOMAC) VA3.1 visual analogue scale (VAS) version. In this version, each item is assessed on a 100-mm VAS, resulting in a maximum cumulative score of 2400 mm. Participants with a total WOMAC score <600 mm, corresponding to less than 25% of the maximum WOMAC score, were considered to have a low symptom burden. WOMAC was used only as an additional clinical screening criterion to reduce the likelihood of including participants with clinically significant OA-related symptoms in the non-OA group and was not included in the OA severity score construction itself. Nevertheless, asymptomatic or undiagnosed OA could not be completely excluded.

Scores for the left and right hip and knee joints were summed, resulting in a theoretical lower-limb OA score ranging from 0 to 8. This score served as an exploratory cumulative OA burden measure reflecting the overall extent of lower-limb OA involvement. Hand osteoarthritis (HOA) was assessed clinically according to the American College of Rheumatology (ACR) criteria [19] and classified dichotomously as absent (0) or present (1). HOA was evaluated separately from the lower-limb OA score in subsequent analyses.

SAF was measured in all participants using the AGE Reader device (DiagnOptics, Groningen, The Netherlands) and was expressed as the ratio of emitted to reflected light in arbitrary units (AU). Three consecutive measurements were taken on both forearms, and the average value was used for the analysis. All measurements were performed in a dark room under standardized conditions to ensure consistency and operator independence, thereby ensuring high reproducibility.

A total of 404 participants were included in the final analysis, including a subset of 48 patients scheduled for elective orthopedic surgery who agreed to provide additional biological samples for biochemical analysis of AGEs and sRAGE. Patients undergoing total knee arthroplasty due to advanced knee OA additionally provided synovial fluid samples collected intraoperatively prior to prosthesis implantation, whereas control participants undergoing other orthopedic procedures without clinical or radiographic evidence of OA provided peripheral blood samples only. Peripheral venous blood was collected during fasting on the morning of surgery into EDTA tubes and centrifuged using an Eppendorf 5702 centrifuge (Eppendorf, Hamburg, Germany) at 1000 × *g* for 15 min to obtain plasma.

Synovial fluid samples were aspirated intraoperatively before prosthesis implantation, centrifuged to remove cellular debris, and processed according to the manufacturer’s recommendations prior to analysis. All plasma and synovial fluid samples were aliquoted and stored at −70 ^∘^C until biochemical analysis.

After reviewing medical documentation and re-evaluating eligibility criteria, 43 participants were deemed eligible for the biochemical subgroup. One OA participant was excluded from the analysis due to insufficient material, resulting in a final analytical sample of 42 participants (21 OA patients and 21 controls). Paired plasma–synovial fluid analyses were performed in 21 OA patients with available synovial fluid samples.

Quantification of AGEs and sRAGE was conducted at the Institute of Pathophysiology, Faculty of Medicine, University of Ljubljana, using validated commercial enzyme-linked immunosorbent assay (ELISA) kits (Abcam, Cambridge, UK). AGEs levels were assessed indirectly by quantifying Nɛ-(carboxymethyl)lysine (CML) using the AGE Assay Kit (ab238539). Soluble RAGE (sRAGE) concentrations were measured using the Human RAGE SimpleStep ELISA Kit (ab190807, Abcam, Cambridge, UK). Absorbance measurements were performed using a Victor 1420-050 spectrophotometer (PerkinElmer, Waltham, MA, USA). All samples were measured in duplicate according to the manufacturers’ instructions. Final concentrations were calculated by interpolation from standard calibration curves. Laboratory analyses were performed using anonymized coded samples, and laboratory personnel were blinded to the clinical status and OA classification of participants during biomarker analyses.

### Ethical statement

The study protocol was approved by the National Medical Ethics Committee of the Republic of Slovenia (approval number 0120-46/2021/4), and written informed consent was obtained from all participants. The study adhered to the Declaration of Helsinki.

### Statistical analysis

Statistical analyses were performed using IBM SPSS Statistics for Windows, Version 29.0 (IBM Corp., Armonk, NY, USA) and GraphPad Prism version 10.1.2 (GraphPad Software, Boston, MA, USA). The intraclass correlation coefficient (ICC) was used to assess the reproducibility of SAF measurements.

Collinearity among predictors and their interactions was assessed prior to modeling. Age and BMI were centered to mitigate potential multicollinearity and enhance the interpretability of interaction terms. Interaction terms (age×BMI_c, age×gender_c, gender×smoking_c, and BMI×smoking_c) were included in the model.

Ordinal logistic regression was employed to examine the association between predictors and lower-limb OA burden score. Binary logistic regression was used to assess factors associated with the presence of HOA. Spearman correlation was utilized to examine relationships between AGEs and sRAGE concentrations in different biological compartments. Independent-samples *t*-tests were used to compare continuous variables between OA patients and controls, while paired *t*-tests compared plasma and synovial fluid concentrations of AGE (CML) and sRAGE. A *P*-value < 0.05 was considered statistically significant.

## Results

### Skin AGEs and OA

A total of 421 outpatients were initially recruited for SAF measurements using the AGE Reader device. After excluding 17 individuals based on exclusion criteria and incomplete data, the final sample comprised 404 participants (179 men and 225 women). Detailed demographic characteristics are presented in Table 1.

**Table 1:** Demographic and clinical characteristics and distribution of hip and knee OA severity scores by gender (*n* ═ 404)

**Variable**	**Men (*n* ═ 179)**	**Women (*n* ═ 225)**	**Total (*n* ═ 404)**
Mean age (years)	64.51 ± 5.78	64.87 ± 5.51	64.71 ± 5.63
Mean SAF (AU)	2.30 ± 0.48	2.26 ± 0.43	2.28 ± 0.45
Mean BMI (kg/m^2^)	28.40 ± 4.30	28.53 ± 5.21	28.47 ± 4.82
Smokers, *n*, (%)	37 (20.7%)	50 (22.2%)	87 (21.5%)
OA severity score, n, (%)			
Score 0	89 (49.7%)	100 (44.4%)	189 (46.8%)
Score 1	13 (7.3%)	23 (10.2%)	36 (8.9%)
Score 2	50 (27.9%)	47 (20.9%)	97 (24.0%)
Score 3	8 (4.5%)	19 (8.4%)	27 (6.7%)
Score 4	18 (10.1%)	31 (13.8%)	49 (12.1%)
Score 5	0 (0.0%)	3 (1.3%)	3 (0.7%)
Score 6	1 (0.6%)	2 (0.9%)	3 (0.7%)

Values are presented as mean ± standard deviation or n (%). OA severity scores in the present cohort were based on clinical and available radiographic assessments of hip and knee osteoarthritis and ranged from 0 (no OA) to 6 (severe OA). **Abbreviations**: AU, arbitrary units; BMI, body mass index; OA, osteoarthritis; SAF, skin autofluorescence.

**Figure 1.**
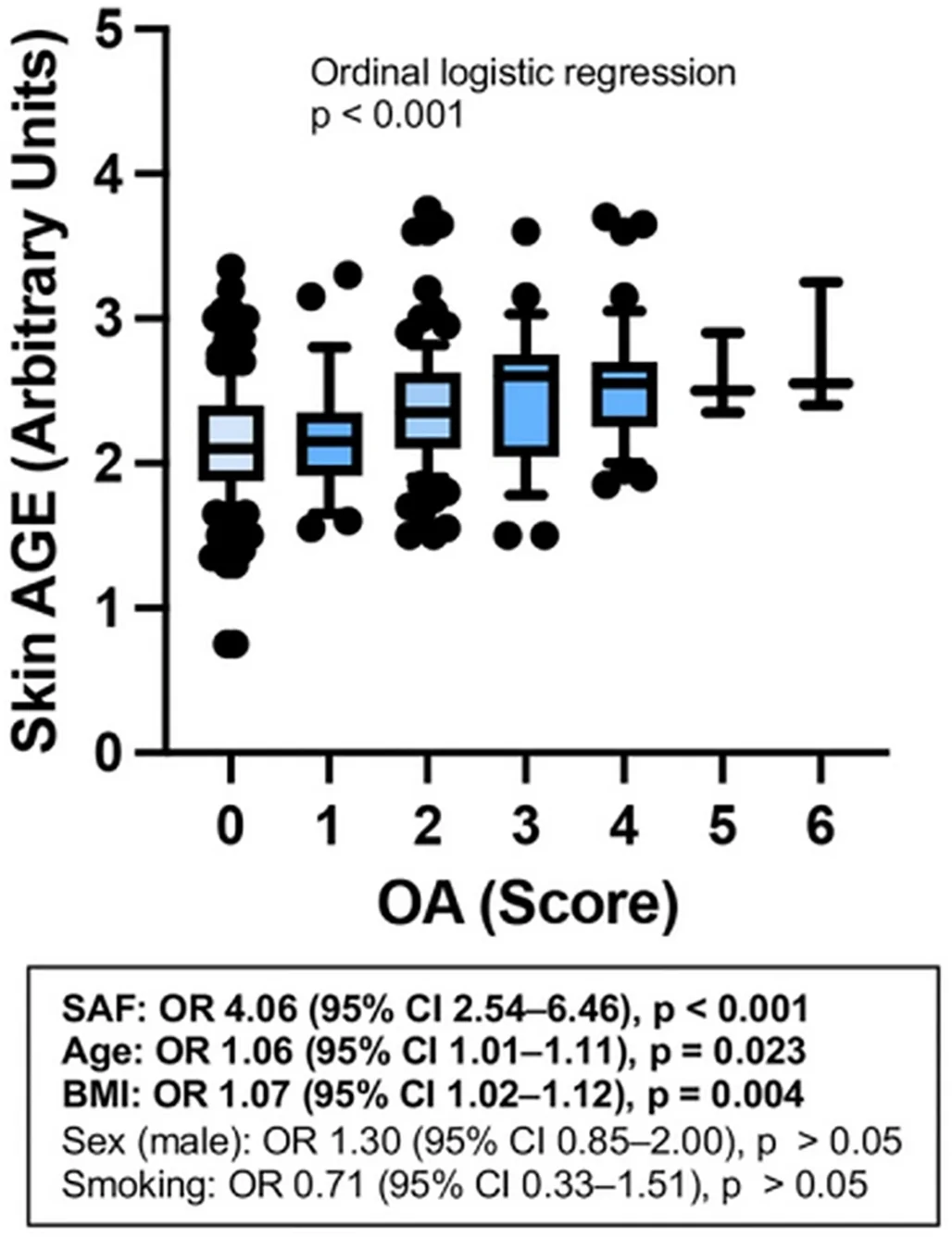
**Association between SAF — a measure of skin AGE levels — and lower-limb OA severity (*n* ═ 404).** Data are presented as boxplots with individual observations. A statistically significant positive association between increasing lower-limb OA severity and SAF was observed in the multivariable ordinal logistic regression model (*P <* 0.001). Regression results are adjusted for age, sex, BMI, and smoking. **Abbreviations**: AGE, advanced glycation end product; BMI, body mass index; OA, osteoarthritis; SAF, skin autofluorescence.

The mean SAF score was 2.30 (SD = 0.48) for men and 2.26 (SD = 0.43) for women. The average age was 64.71 years (SD = 5.63), with a similar distribution between men and women. The mean BMI was 28.47 kg/m^2^ (SD = 4.82), also comparable between sexes, with a slightly wider distribution among women. The proportion of current smokers was 21.5%.

The lower-limb OA burden scores, based on radiographic and clinical assessment of hip and knee joints, are summarized in Table 1. Of all participants, 46.8% exhibited no clinical or radiological signs of OA (grade 0), while 8.9% had mild OA (grade 1), 24.0% had moderate OA (grade 2), and 6.7% had advanced OA (grade 3). An additional 12.1% were classified as having severe OA (grade 4), whereas grades 5 and 6 were rare, each observed in only 0.7% of the population. Women had a slightly higher proportion of grade 3 and 4 OA compared to men. The theoretical lower-limb OA score ranged from 0–8; however, in the present cohort, the highest observed OA severity score was 6, resulting in an observed score range of 0–6.

First, the association between skin AGE and increasing OA severity score was examined using ordinal logistic regression (Figure 1). The distribution of OA involvement across individual joint regions is summarized in Table 2. Median SAF values increased with increasing OA severity score, although the increase appeared less pronounced at OA scores above 3. After adjustment for age, sex, smoking, and BMI, SAF demonstrated a strong and statistically significant positive association with lower-limb OA severity (OR 4.06; 95% CI 2.54–6.46; *P <* 0.001). Age was also significantly associated with higher OA scores (OR 1.06; 95% CI 1.01–1.11; *P ═* 0.023), as was BMI (OR 1.07; 95% CI 1.02–1.12; *P ═* 0.004), albeit with smaller effect sizes. Conversely, gender and smoking were not significantly associated with lower-limb OA severity.

**Table 2:** Distribution of hip and knee OA by joint region and severity grade

**Joint region**	**Grade 1, *n***	**Grade 2, *n***
Left knee OA	13	21
Right knee OA	18	10
Left hip OA	3	17
Right hip OA	2	19

Grade 1 indicates mild-to-moderate OA, and grade 2 indicates severe OA. Participants could have OA involvement in multiple joint regions; therefore, the categories are not mutually exclusive. **Abbreviation**: OA, osteoarthritis.

### SAF and OA in hand joints

Next, the presence of OA in non-weight-bearing joints and its association with AGE accumulation in the skin was investigated (Figure 2). A total of 200 participants had HOA, including 77 with isolated hand OA involvement. Information regarding erosive hand OA was not systematically collected. Participants with HOA had significantly higher SAF scores, and their characteristics are summarized in Table 3. On average, HOA participants were older and had a slightly lower BMI than those without HOA.

**Figure 2.**
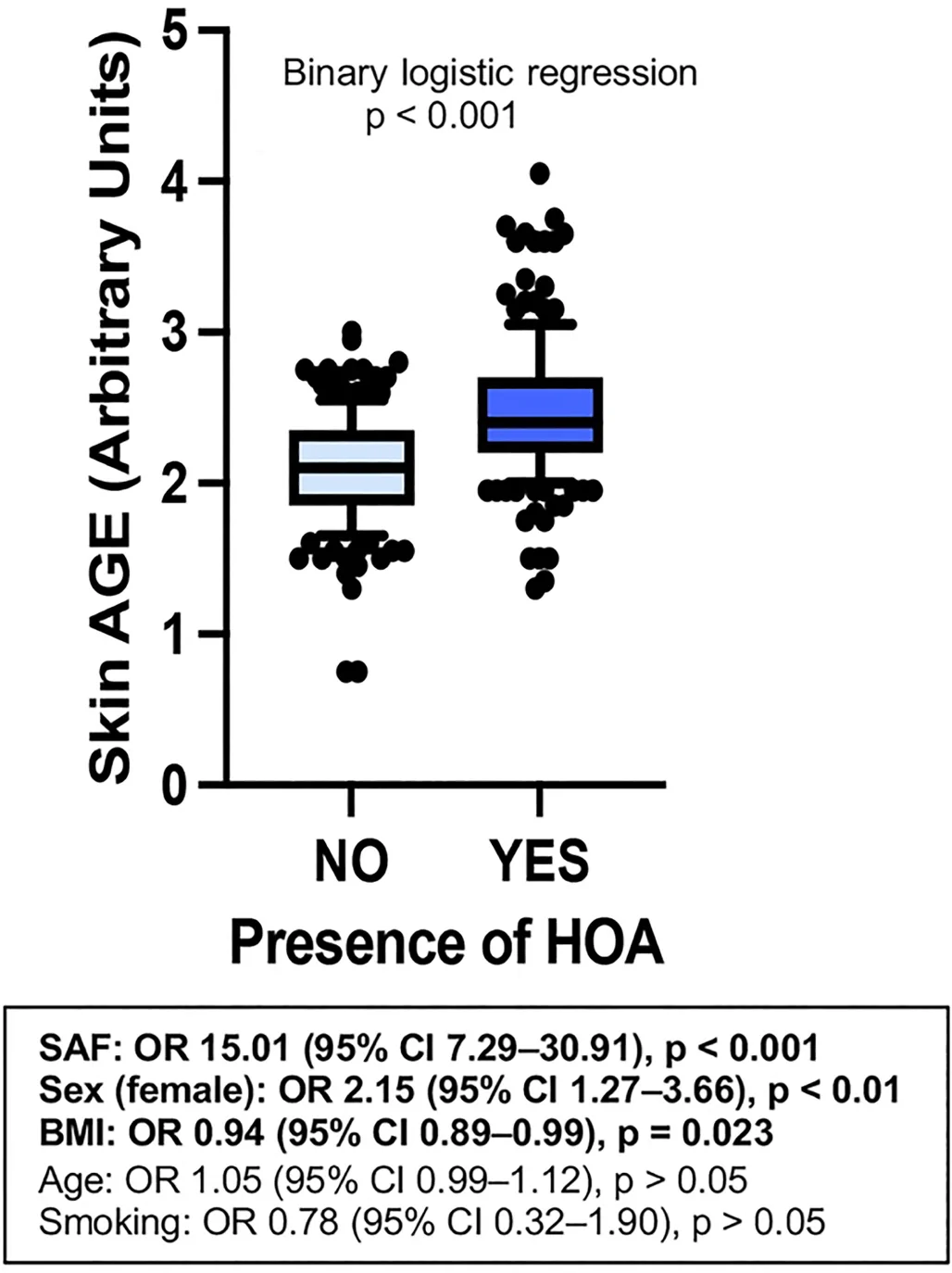
**Association between SAF-measured skin AGE levels and HOA presence.** Data are presented as boxplots with individual observations for participants with and without HOA (*n* ═ 404). Higher SAF values were significantly associated with the presence of HOA in the multivariable binary logistic regression model (*P <* 0.001). Regression estimates shown below the plot were adjusted for age, sex, BMI, and smoking status. Age is expressed in years, BMI in kg/m^2^, and SAF in AU. Participants without HOA may still have hip and/or knee OA involvement and therefore should not be interpreted as an OA-free control group. **Abbreviations**: AGE, advanced glycation end product; AU, arbitrary units; BMI, body mass index; HOA, hand osteoarthritis; OA, osteoarthritis; SAF, skin autofluorescence.

**Table 3:** Demographic and clinical characteristics by presence of HOA

**Variable**	**HOA Present (*n* ═ 200)**	**HOA Absent (*n* ═ 204)**
Women, *n*, (%)	124 (62.0%)	101 (49.5%)
Men, *n*, (%)	76 (38.0%)	103 (50.5%)
Mean BMI (kg/m^2^)	28.08 ± 4.95	28.85 ± 4.67
Mean age (years)	66.23 ± 5.52	63.23 ± 5.34
Mean SAF (AU)	2.47 ± 0.45	2.09 ± 0.36

Participants in the HOA absent group could still present hip and/or knee OA and therefore did not represent an OA-free control group. Values are presented as mean ± standard deviation or n, (%). **Abbreviations**: AU, arbitrary units; BMI, body mass index; HOA, hand osteoarthritis; OA, osteoarthritis; SAF, skin autofluorescence.

To further investigate factors associated with HOA, a multivariable binary logistic regression model was fitted. The dependent variable was binary HOA status (0 = no HOA, 1 = HOA present), with gender, age, BMI, smoking status, and SAF included as predictors.

SAF was the strongest predictor of HOA (OR = 15.0; 95% CI: 7.29–30.91; *P <* 0.001), confirming a robust and independent effect. Gender was also significantly associated with higher HOA scores (OR = 2.15; 95% CI: 1.27–3.66; *P <* 0.01). In contrast, BMI (centered) showed a modest but significant inverse association with HOA (OR = 0.94; 95% CI: 0.89–0.99; *P ═* 0.0238). Neither age nor smoking was significantly associated with HOA. Including the same interaction terms as in the previous model introduced substantial multicollinearity; however, the main effect of SAF remained strong and stable.

### Analysis of circulating and synovial AGEs

AGEs as biomarkers were further assessed in 42 surgical patients: 21 with clinically confirmed OA (total knee arthroplasty group) and 21 control subjects who underwent surgery for other orthopedic indications. To investigate the involvement of the AGEs–RAGE axis at both systemic and local levels, AGEs (carboxymethyl-lysine) and sRAGE were examined in plasma and synovial fluid. The demographic and clinical characteristics of the OA and control groups are presented in Table 4. Both groups included 2 smokers and 9 participants with HOA. The groups were comparable with respect to demographic characteristics, age (*P ═* 0.164), BMI (*P ═* 0.305), and SAF (*P ═* 0.081). The investigated parameters did not differ significantly between the control and OA groups for plasma CML concentrations (*P ═* 0.937) and plasma sRAGE concentrations (*P ═* 0.582). However, the study may have been underpowered to detect small differences due to the limited sample size, and the presence of HOA in both groups may have reduced between-group contrasts. Supporting this possibility, SAF differed significantly in the larger cohort analyzed previously, whereas the difference did not reach statistical significance in the current subset.

**Table 4:** Demographic and clinical characteristics of participants included in the biochemical subgroup analysis (n ═ 42)

**Variable**	**Controls (*n* ═ 21)**	**OA group (*n* ═ 21)**	***P* value**
Women, *n*, (%)	8 (38.1%)	8 (38.1%)	-
Men, *n*, (%)	13 (61.9%)	13 (61.9%)	-
Smokers, *n*, (%)	2 (9%)	2 (9%)	-
HOA present, *n*, (%)	9 (43%)	9 (43%)	-
Age (years, mean ± SD)	66.05 ± 5.50	68.48 ± 5.61	0.164
BMI (kg/m^2^, mean ± SD)	29.28 ± 4.73	30.99 ± 6.83	0.305
SAF (AU, mean ± SD)	2.21 ± 0.38	2.41 ± 0.35	0.081
Plasma AGE (CML) (mean ± SD)	84.15 ± 23.07	83.48 ± 30.99	0.937
Plasma sRAGE (mean ± SD)	844.74 ± 581.12	743.26 ± 604.08	0.582

The control group did not exhibit clinical or radiographic evidence of advanced knee OA requiring arthroplasty; however, some participants presented hand OA according to the ACR criteria. Therefore, the control group should not be interpreted as an entirely OA-free population. Independent-samples *t*-tests were used to compare continuous variables between OA patients and controls. No statistically significant differences were observed between the control and OA groups for demographic variables or the investigated parameters. The absence of significant differences can be interpreted in the context of the limited sample size and the presence of hand OA in both groups. Values are presented as mean ± standard deviation or n (%). **Abbreviations**: ACR, American College of Rheumatology; AGE, advanced glycation end product; AU, arbitrary units; BMI, body mass index; CML, Nɛ-(carboxymethyl)lysine; HOA, hand osteoarthritis; OA, osteoarthritis; SAF, skin autofluorescence; SD, standard deviation; sRAGE, soluble receptor for advanced glycation end products.

Table 5 summarizes paired comparisons of plasma and synovial fluid biomarker concentrations in surgical OA patients.

**Table 5:** Comparison of plasma and synovial fluid concentrations of AGEs and sRAGE in paired samples from surgical OA patients (*n* ═ 21)

**Variable**	**Plasma (mean ± SD)**	**Synovial fluid (mean ± SD)**	***P* value**
AGE (CML)	83.48 ± 30.99	57.14 ± 47.70	0.038
sRAGE	743.26 ± 604.08	183.83 ± 135.80	<0.001

Synovial fluid samples were available only from surgical OA patients, as aspiration was not performed in control participants without clinical indication. Values are presented as mean ± standard deviation. Paired *t*-tests were used to compare plasma and synovial fluid concentrations. **Abbreviations**: AGEs, advanced glycation end products; CML, Nɛ-(carboxymethyl)lysine; OA, osteoarthritis; SD, standard deviation; sRAGE, soluble receptor for advanced glycation end products.

Spearman correlation analysis revealed important correlations, as shown in the heatmap (Figure 3). Correlations involving synovial biomarkers were based on paired OA samples with available synovial fluid measurements (*n* ═ 21), whereas plasma biomarker correlations were evaluated in participants with valid plasma biomarker measurements (*n* ═ 42). A negative correlation was observed between SAF and synovial sRAGE (*ρ ═* −0.571; *P ═* 0.007), indicating an inverse association. This finding is consistent with the notion that higher tissue AGE accumulation is associated with lower sRAGE levels. An inverse correlation was also observed between plasma AGEs levels and plasma sRAGE (*ρ ═* −0.481; *P ═* 0.027). The concentrations of both AGEs and sRAGE were significantly lower in synovial fluid compared to plasma. AGE concentrations showed a modest but statistically significant decrease (paired *t*-test, *P ═* 0.038), while sRAGE concentrations were markedly lower in synovial fluid compared to plasma (paired *t*-test, *P <* 0.001; Figure 4).

**Figure 3.**
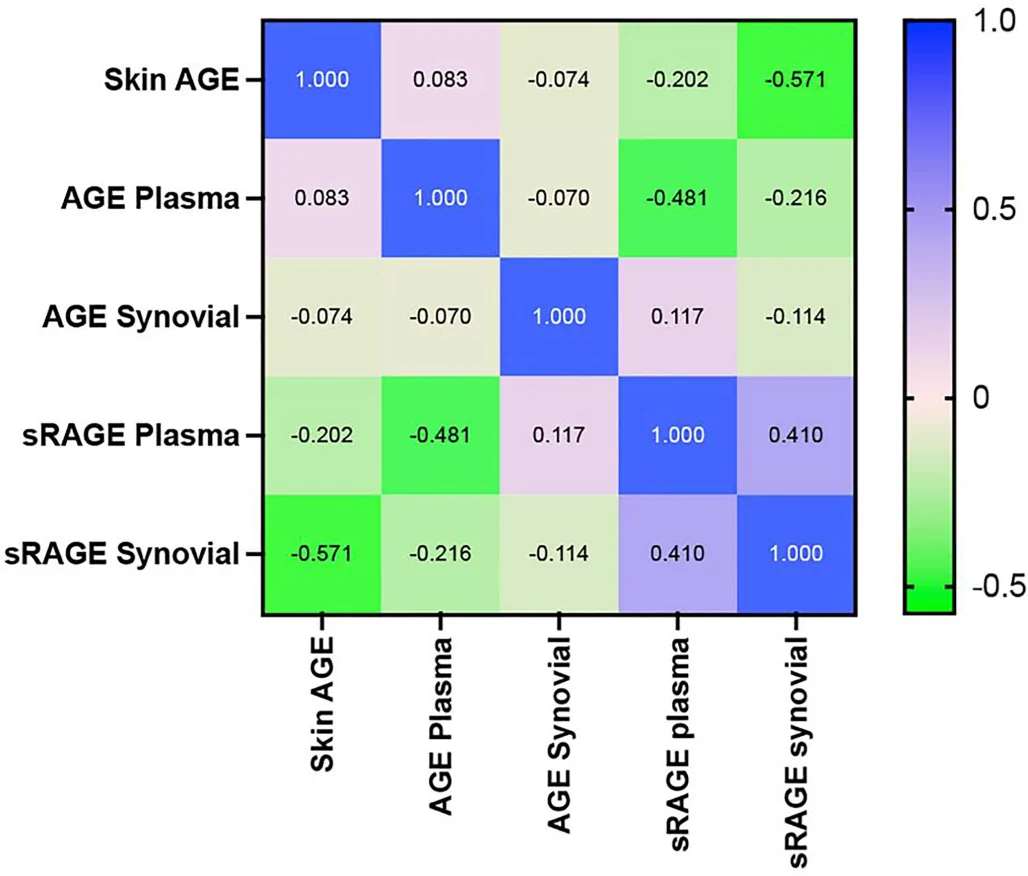
**Correlation heatmap of SAF, AGE, and sRAGE biomarkers in plasma and synovial fluid.** Heatmap showing Spearman correlation coefficients among SAF-measured skin AGE accumulation, plasma and synovial AGE concentrations, and plasma and synovial sRAGE levels. Plasma biomarker correlations were assessed in participants with valid plasma measurements (*n* ═ 42), whereas correlations involving synovial biomarkers were based on paired OA samples with available synovial fluid measurements (*n* ═ 21). Plasma and synovial AGE concentrations were measured as CML by ELISA. The color scale indicates the direction and strength of the correlations, and the corresponding Spearman correlation coefficients are displayed within each cell. **Abbreviations**: AGE, advanced glycation end product; CML, Nɛ-(carboxymethyl)lysine; ELISA, enzyme-linked immunosorbent assay; OA, osteoarthritis; SAF, skin autofluorescence; sRAGE, soluble receptor for advanced glycation end products.

**Figure 4.**
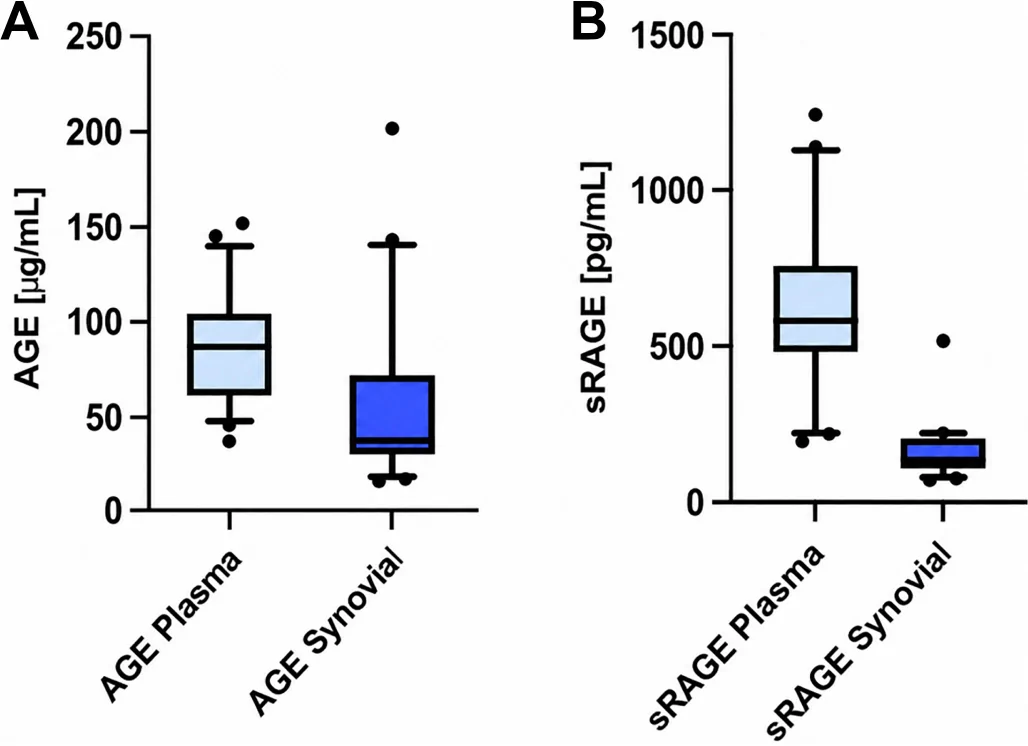
**Plasma and synovial AGE and sRAGE concentrations in surgical OA patients.** Boxplots with individual observations showing paired plasma and synovial fluid biomarker concentrations in surgical OA patients (*n* ═ 21). The left panel compares AGE concentrations measured as CML, and the right panel compares sRAGE concentrations. Synovial fluid concentrations were significantly lower than plasma concentrations for both AGE (*P ═* 0.038) and sRAGE (*P <* 0.001), based on paired *t*-tests. **Abbreviations**: AGE, advanced glycation end product; CML, Nɛ-(carboxymethyl)lysine; OA, osteoarthritis; sRAGE, soluble receptor for advanced glycation end products.

## Discussion

AGEs were investigated as potential underlying factors for the development of OA in a large cohort of outpatients and a subgroup of surgical patients. Both fluorescent and non-fluorescent AGEs were analyzed. SAF, a surrogate marker for fluorescent AGEs accumulation, demonstrated the strongest independent association with OA severity. Other significant predictors included older age and higher BMI. SAF was also strongly associated with OA in the non-weight-bearing joints of both hands. Analysis of carboxymethyl-lysine (a non-fluorescent AGE) and sRAGE in the plasma and synovial fluid of surgical patients revealed lower concentrations of both AGEs and sRAGE in synovial fluid compared to plasma. A negative correlation was observed between AGEs and sRAGE in both plasma and synovial fluid, consistent with the protective role of sRAGE in mitigating inflammatory signaling through membrane-bound AGEs receptors.

Elevated SAF levels in patients with OA, particularly women, align with previous research [20]. However, a reported association between SAF and radiographically defined OA lost significance after excluding participants with joint replacements. This was attributed to the lower prevalence of OA and joint replacement in men, which may have limited statistical power. Conversely, a predictive effect of SAF for knee OA in men, independent of age and BMI, has been previously reported, while no such association was found in women [21]. In the present study, SAF emerged as the strongest independent predictor of lower-limb OA severity, followed by age and BMI, and was also independently associated with hand OA. Several methodological differences, including the higher average age and more balanced gender distribution of our participants, could account for these discrepancies.

Matsumoto et al. [18] found no significant differences in SAF between OA patients and control subjects. Their study had notable limitations, including a small sample size (*n* ═ 24), the inclusion of patients with diverse OA joint sites, and insufficient control for potential confounding factors. In contrast, our study’s larger sample size and well-defined study population facilitated a more reliable assessment of systemic AGEs accumulation and its association with OA severity.

Jiang et al. [22] reported that AGEs accumulation provides a better reflection of long-term exposure to chronic hyperglycemia and inflammation, as AGEs bind to slowly renewable proteins such as collagen. Given that skin collagen has a half-life of approximately 14 years [23, 24], SAF serves as a reliable indicator of chronic AGEs accumulation. Rigalleau et al. [24] further confirmed SAF as a marker of long-term glycation due to slow collagen turnover. Consistent with previous findings, AGEs accumulation increases cartilage stiffness and impairs matrix synthesis, supporting our observation of higher SAF in patients with more severe OA [25]. These results underscore the utility of SAF as a surrogate marker for chronic AGEs burden and its role in OA pathogenesis.

Our findings indicate that age was an independent predictor of higher OA severity. The average age of participants was 64.71 years (range 55–75). This age range was intentionally restricted to better isolate age-related effects from other severity-related factors. These findings align with previous studies identifying age as a major risk factor for OA [6, 26]. The prevalence of OA significantly increases with age—from 27% in individuals younger than 70% to 44% in those older than 80 [26], and from 12.9% at 30–40 years to 43.7% in those over 80 years [2]. Aging enhances joint susceptibility to biomechanical stress, injury, genetic predisposition, and obesity [27]. Although the precise molecular mechanisms remain to be fully elucidated, age-related changes in cartilage structure and function contribute to OA onset [28]. Hirsch et al. [29] reported that both the number of affected joints and OA severity increased with age.

Higher BMI was also associated with increased OA severity in our study, with participants having a mean BMI of 28.47 kg/m^2^, suggesting that even overweight (but not obese) individuals are at elevated risk. Excess body weight contributes to OA through both mechanical overload, as knee joint forces can reach 3–6 times body weight during walking [30], and systemic inflammation mediated by adipokines that disrupt joint homeostasis [31]. Epidemiological evidence supports these findings: the Framingham study demonstrated that a 2-unit BMI reduction (approximately 5 kg) reduces the risk of OA by over 50%, particularly in women [32, 33]. Similarly, every 2-unit increase in BMI elevates the risk of knee OA by 36% [34]. BMI remains an independent predictor, irrespective of age [35], as confirmed by the National Health and Nutrition Examination Survey I (NHANES I) study and a systematic review [36]. Furthermore, population data link metabolic syndrome to OA progression [37, 38], reinforcing the multifactorial impact of excess weight.

In our study, men were slightly less likely to have severe OA compared to women. This is consistent with reports of a higher prevalence of knee OA and greater pain and functional limitations in women, even without severe radiographic changes [39]. Segal et al. [40] also found that OA is more common in women due to biological vulnerability and differences in pain perception. Additionally, a meta-analysis of 85 studies reported a higher risk of knee OA in women [36]. The evaluation of additional variables and larger study samples would enable more robust conclusions.

The association between AGEs and OA is well-established in weight-bearing joints and metabolic diseases; however, studies specifically examining SAF and HOA are limited. In our study, SAF was higher in the HOA group compared to controls, suggesting that systemic AGEs accumulation contributes to OA even in non-weight-bearing joints. In contrast to previously published data [41, 42], age was not a significant predictor in our model. This discrepancy is likely attributable to the age-restricted nature of our study population, which may have reduced age variability and consequently its statistical impact.

Women were more prevalent in our HOA group (62.0% vs. 49.5%), and multivariable analysis showed that female gender was associated with a 2.15-fold higher likelihood of having hand OA. This observation aligns with previous radiological and population-based studies demonstrating a higher prevalence of hand OA among women [43] and supports earlier findings reporting radiographic hand OA in at least one joint in 67% of women and 54.8% of men [44]. More recent evidence also suggests that hormonal factors may partly contribute to the greater susceptibility of women to hand OA [45].

Given the key role of AGEs in joint degeneration, we focused on carboxymethyl-lysine as a representative marker due to its systemic distribution, RAGE-activating potential, and feasibility of analysis by ELISA. We also included sRAGE and SAF to obtain a broader view of systemic AGEs accumulation. SAF primarily detects fluorescent AGEs, such as pentosidine, and its levels have also been shown to correlate with the tissue content of non-fluorescent AGEs, including carboxymethyl-lysine and carboxyethyl-lysine, as assessed in skin biopsies [46]. We were unable to confirm the correlations between SAF and carboxymethyl-lysine in plasma or synovial fluid, possibly because fluorescent and non-fluorescent AGEs were measured in different tissues. In contrast, a negative correlation was observed between SAF and synovial sRAGE, and between plasma carboxymethyl-lysine and plasma sRAGE, indicating an inverse relationship between AGEs accumulation and sRAGE availability both locally and systemically. These data are consistent with findings showing increased levels of fluorescent AGEs in macroscopically healthy cartilage from OA donors associated with functional RAGE signaling [47]. Reduced sRAGE levels have been observed in both plasma and synovial fluid of patients with knee OA, and these levels are inversely correlated with disease severity [25]. Similarly, our data support the idea that the accumulation of AGEs may decrease the availability of sRAGE, thereby diminishing its protective role. This interpretation is corroborated by findings highlighting the role of sRAGE as a decoy receptor that reduces ligand binding to membrane-bound RAGE, consequently downregulating inflammatory and catabolic gene expression [48]. These conclusions, based on preclinical models and human cartilage explants, are consistent with the negative correlations we observed between carboxymethyl-lysine and sRAGE in plasma and between SAF and sRAGE in synovial fluid.

In contrast to the observed correlations with sRAGE, plasma carboxymethyl-lysine levels were not significantly associated with the assessed OA-related clinical parameters. These findings are consistent with those of Cambon-Binder et al. [49], who reported elevated levels of carboxymethyl-lysine in OA cartilage but no correlation with serum concentration or clinical manifestations. Given the variable circulating levels of sRAGE and its role as a decoy receptor, interpretation remains complex. Erusalimsky et al. [50] cautioned against overinterpretation of sRAGE associations in clinical studies and emphasized the need for further investigation of AGEs–RAGE dynamics in OA pathogenesis.

As with any study, several limitations warrant acknowledgment. SAF reflects total fluorescent AGE accumulation without compound-specific differentiation. Carboxymethyl-lysine, representing only one AGE compound, was measured in a relatively small biochemical subgroup of 21 OA patients and 21 control participants, potentially reducing the precision of estimated associations. Recruitment for this subgroup occurred during the post-COVID period, where reduced surgical volume and limited patient availability further constrained subgroup size. Synovial fluid sampling, dependent on intraoperative availability during total knee arthroplasty and sufficient fluid collection, may have introduced selection bias.

Biochemical analyses were performed on a pooled surgical cohort encompassing both OA and control participants, which may have obscured potential group-specific associations and introduced biological heterogeneity. Furthermore, the control subgroup was not entirely free of OA, as some participants met the clinical criteria for HOA, although the number of participants with HOA was equal in both groups. HOA diagnosis relied on clinical ACR criteria without systematic radiographic confirmation, and erosive HOA was not specifically assessed, potentially affecting diagnostic precision.

Additionally, radiographic imaging was unavailable for all asymptomatic participants. Although supplementary clinical screening criteria were applied, asymptomatic or undiagnosed OA could not be entirely excluded. Finally, the cross-sectional design precludes causal inference, and residual confounding, inherent to observational clinical studies, may still be present.

Despite these limitations, this study is among the first to assess the AGEs–RAGE axis systemically and locally in OA by combining SAF with AGE biomarker measurements in both plasma and synovial fluid. The inclusion of synovial sRAGE measurements further enhances our understanding of the local and systemic processes potentially involved in OA pathophysiology. These findings offer additional insight into the potential relationship between AGE accumulation and OA burden.

SAF was associated with clinical indicators of OA in a large population encompassing both weight-bearing and hand joints, supporting a relationship between systemic AGE accumulation and overall OA burden. The observed associations between SAF, age, BMI, and OA severity further support the concept of a metabolic component in OA pathogenesis and the possibility of polyarticular involvement. While the non-invasive nature of SAF makes it an attractive research tool for assessing long-term AGE accumulation, further longitudinal and diagnostic studies are necessary to determine its potential clinical utility and its role in OA assessment. These findings contribute to the growing evidence supporting the involvement of AGEs in OA pathophysiology and provide a basis for future mechanistic and clinical investigations.

## Conclusion

Skin autofluorescence, a marker of AGE accumulation, was independently and positively associated with overall OA severity after adjusting for age, gender, smoking, and BMI. Higher SAF values were also observed in participants with hand OA, supporting a link between systemic AGE burden and joint involvement. The inverse relationships between AGEs and sRAGE in plasma, as well as between SAF and synovial sRAGE, suggest a potential imbalance between AGE accumulation and protective receptor mechanisms. Collectively, these findings support a role for AGEs in OA pathophysiology and highlight the observed association between SAF and OA burden. While SAF may represent a potential non-invasive biomarker of disease burden, further longitudinal and diagnostic studies are needed to establish its clinical applicability.

## Data Availability

The data supporting the findings of this study are available from the corresponding author upon reasonable request. The data are not publicly available due to ethical and privacy restrictions related to patient confidentiality.

## References

[1] Zhang Y, Zhou Y (2024). Advances in targeted therapies for age-related osteoarthritis: a comprehensive review of current research. Biomed Pharmacother.

[2] He Y, Li Z, Alexander PG, Ocasio-Nieves BD, Yocum L, Lin H (2020). Pathogenesis of osteoarthritis: risk factors, regulatory pathways in chondrocytes, and experimental models. Biology (Basel).

[3] Pettenuzzo S, Berardo A, Belluzzi E, Pozzuoli A, Ruggieri P, Carniel EL (2025). Mechanical insights into fat pads: a comparative study of infrapatellar and suprapatellar fat pads in osteoarthritis. Connect Tissue Res.

[4] Franz A, Joseph L, Mayer C, Harmsen JF, Schrumpf H, Fröbel J (2018). The role of oxidative and nitrosative stress in the pathology of osteoarthritis: novel candidate biomarkers for quantification of degenerative changes in the knee joint. Orthop Rev (Pavia).

[5] Vincent TL, McClurg O, Troeberg L (2022). The extracellular matrix of articular cartilage controls the bioavailability of pericellular matrix-bound growth factors to drive tissue homeostasis and repair. Int J Mol Sci.

[6] Tsujii A, Nakamura N, Horibe S (2017). Age-related changes in the knee meniscus. Knee.

[7] Liu HZ, Song XQ, Zhang H (2024). Sugar-coated bullets: unveiling the enigmatic mystery ‘sweet arsenal’ in osteoarthritis. Heliyon.

[8] Twarda-Clapa A, Olczak A, Białkowska AM, Koziołkiewicz M (2022). Advanced glycation end-products (AGEs): formation, chemistry, classification, receptors, and diseases related to AGEs. Cells.

[9] Goldin A, Beckman JA, Schmidt AM, Creager MA (2006). Advanced glycation end products: sparking the development of diabetic vascular injury. Circulation.

[10] Rouhiainen A, Kuja-Panula J, Tumova S, Rauvala H.

[11] Kalea AZ, Schmidt AM, Hudson BI (2011). Alternative splicing of RAGE: roles in biology and disease. Front Biosci.

[12] Raucci A, Cugusi S, Antonelli A, Barabino SM, Monti L, Bierhaus A (2008). A soluble form of the receptor for advanced glycation endproducts (RAGE) is produced by proteolytic cleavage of the membrane-bound form by the sheddase a disintegrin and metalloprotease 10 (ADAM10). FASEB J.

[13] Oliveira MI, Souza EM, Pedrosa FD, Réa RR, Alves AD, Picheth G (2013). RAGE receptor and its soluble isoforms in diabetes mellitus complications. J Bras Patol Med Lab.

[14] He CP, Chen C, Jiang XC, Li H, Zhu LX, Wang PX (2022). The role of AGEs in pathogenesis of cartilage destruction in osteoarthritis. Bone Joint Res.

[15] Saudek DM, Kay J (2003). Advanced glycation endproducts and osteoarthritis. Curr Rheumatol Rep.

[16] DeGroot J, Verzijl N, Bank RA, Lafeber FP, Bijlsma JW, TeKoppele JM (1999). Age-related decrease in proteoglycan synthesis of human articular chondrocytes: the role of nonenzymatic glycation. Arthritis Rheum.

[17] Lou A, Wang L, Lai W, Zhu D, Wu W, Wang Z (2021). Advanced oxidation protein products induce inflammatory responses and invasive behaviour in fibroblast-like synoviocytes via the RAGE–NF-κB pathway. Bone Joint Res.

[18] Matsumoto T, Tsurumoto T, Baba H, Osaki M, Enomoto H, Yonekura A (2008). Measurement of advanced glycation endproducts in skin of patients with rheumatoid arthritis, osteoarthritis, and dialysis-related spondyloarthropathy using non-invasive methods. Rheumatol Int.

[19] Altman R, Alarcon G, Appelrouth D, Bloch D, Borenstein D, Brandt K (1990). The American College of Rheumatology criteria for the classification and reporting of osteoarthritis of the hand. Arthritis Rheum.

[20] Waqas K, Szilagyi IA, Schiphof D, Boer CG, Bierma-Zeinstra S, van Meurs JBJ (2022). Skin autofluorescence and its relation to radiographic and MRI-based osteoarthritis. Osteoarthritis Cartilage.

[21] Eaton CB, Sayeed M, Ameernaz S, Roberts MB, Maynard JD, Driban JB (2017). Sex differences in the association of skin advanced glycation end-products with knee osteoarthritis progression. Arthritis Res Ther.

[22] Jiang J, Zhang Y, Chen J, Yang X, Mei C, Xiong F (2021). Serum and tissue levels of advanced glycation end products and risk of mortality in patients on maintenance hemodialysis. Am J Nephrol.

[23] Verzijl N, DeGroot J, Thorpe SR, Bank RA, Shaw JN, Lyons TJ (2000). Effect of collagen turnover on the accumulation of advanced glycation end products. J Biol Chem.

[24] Rigalleau V, Pucheux Y, Couffinhal T, Tessier FJ, Howsam M, Rubin S (2024). Skin autofluorescence of advanced glycation end-products, glycemic memory, and diabetes complications. Diabetes Metab.

[25] Chayanupatkul M, Honsawek S (2010). Soluble receptor for advanced glycation end products (sRAGE) in plasma and synovial fluid is inversely associated with disease severity of knee osteoarthritis. Clin Biochem.

[26] Felson DT, Naimark A, Anderson J, Kazis L, Castelli W, Meenan RF (1987). The prevalence of knee osteoarthritis in the elderly. The Framingham Osteoarthritis Study. Arthritis Rheum.

[27] Loeser RF, Collins JA, Diekman BO (2016). Ageing and the pathogenesis of osteoarthritis. Nat Rev Rheumatol.

[28] Li Y, Wei X, Zhou J, Wei L (2013). The age-related changes in cartilage and osteoarthritis. Biomed Res Int.

[29] Hirsch R, Lethbridge-Cejku M, Scott WW, Reichle R, Plato CC, Tobin J (1996). Association of hand and knee osteoarthritis: evidence for a polyarticular disease subset. Ann Rheum Dis.

[30] Bliddal H, Leeds AR, Christensen R (2014). Osteoarthritis, obesity and weight loss: evidence, hypotheses and horizons-a scoping review. Obes Rev.

[31] Francisco V, Pérez T, Pino J, López V, Franco E, Alonso A (2018). Biomechanics, obesity, and osteoarthritis. The role of adipokines: when the levee breaks. J Orthop Res.

[32] Felson DT, Anderson JJ, Naimark A, Walker AM, Meenan RF (1988). Obesity and knee osteoarthritis: the Framingham Study. Ann Intern Med.

[33] Felson DT, Zhang Y, Anthony JM, Naimark A, Anderson JJ (1992). Weight loss reduces the risk for symptomatic knee osteoarthritis in women: the Framingham Study. Ann Intern Med.

[34] March LM, Bagga H (2004). Epidemiology of osteoarthritis in Australia. Med J Aust.

[35] Anderson JJ, Felson DT (1988). Factors associated with osteoarthritis of the knee in the first National Health and Nutrition Examination Survey (HANES I): evidence for an association with overweight, race, and physical demands of work. Am J Epidemiol.

[36] Blagojevic M, Jinks C, Jeffery A, Jordan K (2010). Risk factors for onset of osteoarthritis of the knee in older adults: a systematic review and meta-analysis. Osteoarthritis Cartilage.

[37] Xie DX, Wei J, Zeng C, Yang T, Li H, Wang YL (2017). Association between metabolic syndrome and knee osteoarthritis: a cross-sectional study. BMC Musculoskelet Disord.

[38] Yoshimura N, Muraki S, Oka H, Kawaguchi H, Nakamura K, Akune T (2011). Association of knee osteoarthritis with the accumulation of metabolic risk factors: the ROAD study. J Rheumatol.

[39] Szilagyi IA, Waarsing JH, van Meurs JB, Bierma-Zeinstra SM, Schiphof D (2023). A systematic review of the sex differences in risk factors for knee osteoarthritis. Rheumatology (Oxford).

[40] Segal NA, Nilges JM, Oo WM (2024). Sex differences in osteoarthritis prevalence, pain perception, physical function and therapeutics. Osteoarthritis Cartilage.

[41] Kalichman L, Hernández-Molina G (2010). Hand osteoarthritis: an epidemiological perspective. Semin Arthritis Rheum.

[42] Jonsson H (2017). Age-related prevalence of hand osteoarthritis diagnosed by photography (HOASCORE). BMC Musculoskelet Disord.

[43] Ouellette EA, Makowski AL (2006). How men and women are affected by osteoarthritis of the hand. Orthop Clin North Am.

[44] Wilder FV, Barrett JP, Farina EJ (2006). Joint-specific prevalence of osteoarthritis of the hand. Osteoarthritis Cartilage.

[45] Dahaghin S, Bierma-Zeinstra SMA, Ginai AZ, Pols HAP, Hazes JMW, Koes BW (2005). Prevalence and pattern of radiographic hand osteoarthritis and association with pain and disability: the Rotterdam Study. Ann Rheum Dis.

[46] Gulati M, Brewer G, Judge A, Kennedy D, Vincent TL, Watt FE (2024). Could sex-specific subtypes of hand osteoarthritis exist? A retrospective study in women presenting to secondary care. Front Pain Res (Lausanne).

[47] Steenvoorden MM, Huizinga TW, Verzijl N, Bank RA, Ronday HK, Luning HA (2006). Activation of receptor for advanced glycation end products in osteoarthritis leads to increased stimulation of chondrocytes and synoviocytes. Arthritis Rheum.

[48] Millerand M, Berenbaum F, Jacques C (2019). Danger signals and inflammaging in osteoarthritis. Clin Exp Rheumatol.

[49] Cambon-Binder A, Jaisson S, Tuffet S, Courties A, Eymard F, Okwieka A (2023). Serum carboxymethyllysine concentration is associated with erosive hand osteoarthritis. Osteoarthritis Cartilage.

[50] Erusalimsky JD (2021). The use of the soluble receptor for advanced glycation-end products (sRAGE) as a potential biomarker of disease risk and adverse outcomes. Redox Biol.

